# CoVO_3_ High‐Pressure Polymorphs: To Order or Not to Order?

**DOI:** 10.1002/advs.202307766

**Published:** 2023-12-16

**Authors:** Elena Solana‐Madruga, Olivier Mentré, Alexander A. Tsirlin, Marielle Huvé, Dmitry Khalyavin, Clemens Ritter, Angel Moisés Arévalo‐López

**Affiliations:** ^1^ UMR‐8181‐UCCS‐Unité de Catalyse et Chimie du Solide Univ. Lille CNRS Centrale Lille ENSCL Univ. Artois Lille F‐59000 France; ^2^ Dpto. Química Inorgánica Universidad Complutense de Madrid Avda. Complutense sn Madrid 28040 Spain; ^3^ Felix Bloch Institute for Solid‐State Physics Leipzig University 04103 Leipzig Germany; ^4^ ISIS Facility Rutherford Appleton Laboratory Harwell, Didcot Oxford OX11 0QX UK; ^5^ Institut Laue‐Langevin 71 Avenue des Martyrs, Cedex Grenoble 32042 France

**Keywords:** cation ordering, high‐pressure phases, magnetism

## Abstract

Materials properties are determined by their compositions and structures. In ABO_3_ oxides different cation orderings lead to mainly perovskite‐ or corundum like derivatives with exciting physical properties. Sometimes, a material can be stabilized in more than one structural modification, providing a unique opportunity to explore structure‐properties relationship. Here, CoVO_3_ obtained in both ilmenite‐(CoVO_3_‐I) and LiNbO_3_‐type (CoVO_3_‐II) polymorphs at moderate (8–12 GPa) and high pressures (22 GPa), respectively are presented. Their distinctive cation distributions affect drastically the magnetic properties as CoVO_3_‐II shows a cluster‐glass behavior while CoVO_3_‐I hosts a honeycomb zigzag magnetic structure in the cobalt network. First principles calculations show that the influence of vanadium is crucial for CoVO_3_‐I, although it is previously considered as non‐magnetic in a dimerized spin‐singlet state. Contrarily, CoVO_3_‐II shows two independent interpenetrating antiferromagnetic Co‐ and ferromagnetic V‐hcp sublattices, which intrinsically frustrate any possible magnetic order. CoVO_3_‐II is also remarkable as the first oxide crystallizing with the LiNbO_3_‐type structure where both metals contain free *d* electrons. CoVO_3_ polymorphs pinpoint therefore as well to a much broader phase field of high‐pressure A‐site Cobaltites.

## Graphical abstract

Here, the unique diversity of magnetic behavior in two polymorphs of CoVO_3_ is reported. (I) shows a close relation to Kitaev honeycomb magnets with the zigzag‐type magnetic structure and its origin is elucidated using first‐principle calculations. (II) crystallizes in a LiNbO_3_‐type structure and presents a cluster spin glass driven by a novel mechanism of magnetic frustration from interpenetrating antiferromagnetic and ferromagnetic sublattices.

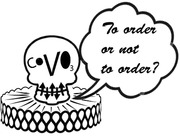

## 1 Introduction

Polymorphism in ABO_3_ oxides often yields to perovskite and corundum‐related structures. Megaw first showed that the LiNbO_3_‐type (LN) structure is related to that of ideal perovskite and predicted that a phase transition in between them could take place with temperature.^[^ ^1^ ^]^ In the field of earth‐ and high‐pressure science, LN‐type compounds are considered to be decompression products of high‐pressure perovskite‐phases, as such several LN‐type oxides like MnMO_3_ (M = Ti, Sn), FeMO_3_ (M = Ti, Ge), MgMO_3_ (M = Ti, Ge), ZnGeO_3_ and CuTaO_3_ have been reported as metastable quenched phases.^[^ ^2^, ^3^, ^4^, ^5^, ^6^, ^7^ ^]^

In the last decade, high‐pressure and high‐temperature synthesis conditions (HP‐HT) provided access to a large family of compounds, where the A site can be occupied by a small cation as Mn^2+^.^[^ ^8^, ^9^ ^]^ Accordingly, simple MnVO_3_ can be obtained in a lower pressure (<3.5 GPa) distorted ilmenite‐type and a higher‐pressure (>4 GPa) perovskite‐type polymorph,^[^ ^10^, ^11^, ^12^ ^]^ whereas MnTiO_3_ recoils from a perovskite‐ to a LN‐type structure under decompression.^[^ ^13^, ^14^ ^]^ Following the same idea other small A cations can be squeezed into these structures as shown by the LN‐type FeTiO_3_ with multiferroic properties.^[^ ^15^ ^]^ Very recently, CoVO_3_‐I was obtained at 8 GPa in an ilmenite phase distorted due to V–V dimerization and exhibiting an antiferromagnetic transition at low temperature.^[^ ^16^ ^]^

This sets a challenge of obtaining a higher‐pressure polymorph in either a perovskite‐ or a LN‐type structure. Here, we report the synthesis of CoVO_3_‐II, the first LN‐type oxide with both magnetic cations, and a comparison of its magnetic properties against those of its low‐pressure polymorph. The discovery of both polymorphs suggests that a broad range of novel A‐site cobaltites may be accessible at HP‐HT conditions.

## 2 Results and Discussion

**Figure 1:**
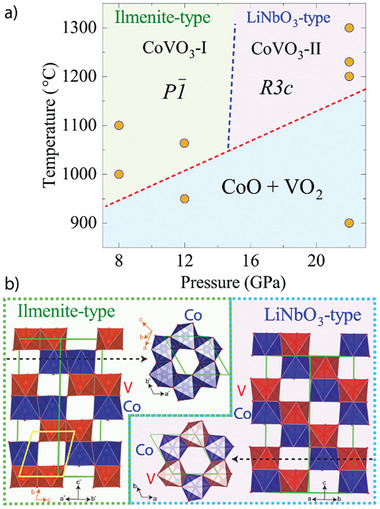
a) “CoO+VO_2_” transformation diagram. b) Comparison of both CoVO_3_ polymorphs in the rhombohedral setting.

We examined the behavior of a mixture of CoO and VO_2_ at HP‐HT conditions in a multianvil apparatus and studied the recovered samples *ex situ*. The “CoO + VO_2_” transformation diagram shown in **Figure** **1**a summarizes the conditions used to obtain the different CoVO_3_ polymorphs. In the 8–12 GPa pressure range, CoVO_3_‐I crystallizes in a distorted ilmenite structure with *a* = 4.9999(4) Å, *b* = 5.4353(4) Å, *c* = 4.9463(5) Å, α = 90.10(1) °, β = 119.79(1) °, and γ = 63.40(1) ° in the *P‐1* space group. The driving force for the *R‐3* → *P‐1* distortion occurring at 550K was reported to be V–V dimerization in the V honeycomb layer, see ref. [15], Figure 1b, and Supporting Information. However, one could not discard that the nearly degenerate 3*t_2g_* levels suffer a Jahn–Teller instability (JT) and the partial dimerization only occurs as a side effect, as observed for instance in BaFe_2_(PO_4_)_2_.^[^ ^16^ ^]^ At 300 K the short V–V distance of 2.7353(3) Å compared to the two other longer ones of 3.03609(3) Å and 3.01466(3) Å suggests direct metal‐metal bonding, which will affect the magnetic properties as discussed below.

Regarding the higher‐pressure CoVO_3_‐II polymorph (obtained at 22 GPa and 1473 K), its crystal structure was solved from a small single crystal (CCDC 2 300 093 and Supporting Information). We found that CoVO_3_‐II is the first oxide that crystallizes in the LiNbO_3_‐type structure containing both A and B transition metal ions with free *d* electrons. It presents *a* = 5.008(5) Å and *c* = 13.54(1) Å cell parameters in the polar *R3c* space group and it is ≈1.2% denser than CoVO_3_‐I at ambient pressure. BVS calculations return values of +2.1(2) for Co and +4.0(4) for V and oxygen octahedra volumes of 11.38 and 9.75 Å^3^, respectively, corroborating the assigned charges. Both structures are compared in Figure 1b. The main difference is clearly seen in the rhombohedral setting, with (*a*’,*b*’,*c*’)*_R_* = (*a*,*b*,*c*)*_T_* [0 0 1, ‐1 0 ‐1, ‐2 3 ‐1] transformation matrix between the rhombohedral (*R*) and the triclinic (*T*) cells. CoVO_3_‐I shows alternating honeycomb layers of either Co or V stacked along the *c* axis in a rhombohedral setting, whereas in CoVO_3_‐II these layers contain both Co and V.

**Figure 2:**
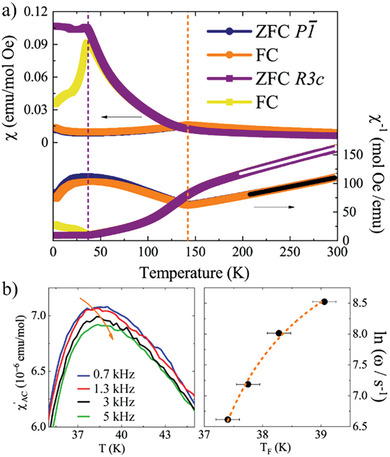
a) Direct and inverse magnetic susceptibility for both CoVO_3_ polymorphs. Curie–Weiss laws fitted at high temperatures are shown with lines. b) Real part of AC‐susceptibility along with the T_F_ change fitted with the Vogel–Fulcher equation for CoVO_3_‐II.

The effects of the different cation orderings on the magnetic properties of CoVO_3_ were explored by comparing both polymorphs as shown in **Figure** **2**a. Magnetic susceptibility measurements reveal that both samples are Curie–Weiss paramagnets in the 150–300 K range, with effective paramagnetic moments of *µ*_eff_ = 5.01 and 4.48 µ_B_ f.u.^−1^ and Weiss temperatures of *θ* = −45 and −65 K for polymorphs I and II respectively. The moments for both polymorphs are high compared to the expected value (*µ*_theo_ = 4.24 µ_B_) from stoichiometric contributions of the spin only Co^2+^ (S = 3/2) and V^4+^ (S = ½) ions, showing that orbital contributions are present. At low temperatures, both polymorphs exhibit a transition at *T*_N_ = 141 K (CoVO_3_‐I) and 38 K (CoVO_3_ ‐II), displaying a frustration index of *f* = |θ|/*T*_N_ = 1.7 for the latter. NPD data collected at low temperature showed no signature of magnetic ordering for CoVO_3_‐II, see Figure S5 (Supporting Information).

In accordance with this, AC‐magnetic susceptibility on CoVO_3_‐II revealed subsequently a spin‐glass behavior, see Figure 2b. The freezing temperature *T*_f_ varies according to the Vogel‐Fulcher equation (ω = ω_0_ exp(E_a_/k_B_(*T*_f_–*T_0_*)) with ω the frequency, E_a_ the activation energy, k_B_ the Boltzmann constant and *T_0_* the Vogel‐Fulcher temperature). The fitting strongly suggests the formation of a cluster glass with ln(ω_0_/Hz) = 14.0(3) → 1/ ω_0_ = 8.32 × 10^−7^ s, *T*_0_ = 33(1) K and E_a_/k_b_ = 7.3(3) K. This disordered glassy state advocates for some antisite cation disorder, however two NPD data sets from D20 and WISH maon different samples confirm the LiNbO_3_‐type structure, where a sufficient contrast between Co and V prevails due to their different neutron scattering lengths (2.49, −0.38, and 5.8 fm for Co, V, and O respectively). This apparent conflict between the magnetically disordered ground state in a structurally well‐ordered compound is explained by its intrinsic magnetic frustration, as we show below.

**Figure 3:**
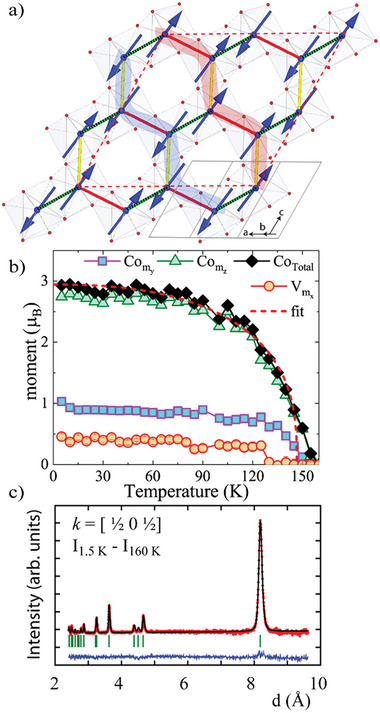
a) CoVO_3_‐I magnetic structure with highlighted zigzag FM chains coupled AFM through the shortest bond (dashed green bond). Only Co atoms are shown. b) Thermal evolution of Co and V magnetic moment adjusted with a critical law as detailed in the text. c) Rietveld refinement of the Intensity difference between 1.5 and 160 K NPD data with *k* = [½ 0 ½].

For CoVO_3_‐I, NPD collected at WISH at low temperatures revealed the appearance of magnetic peaks below *T*_N_. They can be indexed with a *k* = [½ 0 ½] propagation vector. The Rietveld refinement was obtained with Fullprof in the *Ps‐1* (# 2.7) magnetic space group. The model with only Co sublattice yields the moment components [0, −1.11(1), 3.016(9)] µ_B_ at 2 K and the agreement factors *R*_mag_ = 10.52% and χ^2^ = 1.42. Refinement with a finite moment on the V‐sublattice converges with a better agreement factors (*R*_mag_ = 8.64, χ^2^ = 1.05) and the moment components [0.46(1), 0, 0] µ_B_. The model, however, implies nearly orthogonal alignment of the Co and V moments, demolishing their interaction. It is possible that a tiny amount of strongly anisotropic Co^2+^ takes the V‐site and defines the moment direction of this sublattice. Alternatively, the obtained V moment can be an artifact of the under‐constrained model. Although our DFT calculations indirectly support the magnetic nature of the V‐sublattice, as specified below, more precise future experimental measurements are required to confirm this conclusion. For instance, XMCD measurements on both Co and V L_2,3_ edges would unambiguously determine the presence of an intrinsic magnetic moment on V and the relative alignment of this with the Co magnetic moment. This, however, would imply that the V–V dimerization is not complete, that is, a *S* = 0 spin singlet would not exist but only JT distortion, and that some of the V moment still contributes to the magnetic ordering. The vanadium moments are perpendicular to the zigzag‐type magnetic structure defined by the cobalt atoms, plausibly to avoid frustration, see **Figure** **3**a, similar to MnVO_3_‐I where the Mn and V spins are also orthogonal to each other.^[^ ^11^ ^]^ The simultaneous contribution of V *d*‐electrons to both ordered magnetic moments and molecular orbital formation has been previously observed in GaV_4_O_8_ with the so‐called “hybrid‐electrons”.^[^ ^17^ ^]^ The temperature evolution of the Co and V moments, Figure 3b, follows a critical law *µ*(T) = *µ*_0_[1‐[T/*T_N_*]^β^ with *µ*_0_ = 2.94(4) µ_B_ for Co and 0.38(2) µ_B_ for V, *T*_N_ = 147(1) K and *β* = 0.35(3) which is in agreement to the theoretical value of *β* = 0.367 for a 3D Heisenberg magnet, appropriate for the structure described above.

**Figure 4:**
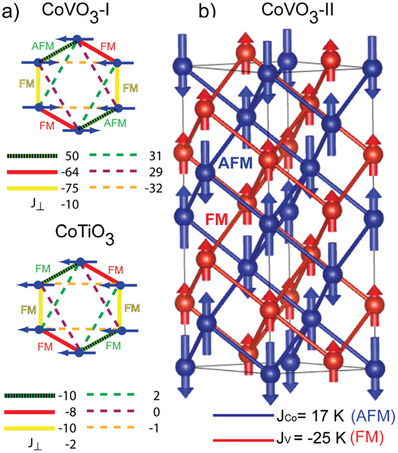
a) Calculated magnetic structure for the honeycomb network of Co atoms in CoVO_3_‐I with their exchange interactions *J* in K. Ignoring the V contribution results in the CoTiO_3_ magnetic structure. b) Calculated magnetic structure for CoVO_3_‐II with the interpenetrating magnetic sublattices of Co^2+^ (AFM) and V^4+^ (FM).

The zigzag‐type order within honeycomb planes is common for Kitaev magnets, such as Na_3_CoSb_2_O_6_, Na_2_IrO_3_, and α‐RuCl_3_ where this spin arrangement is the result of a competition between different exchange couplings.^[^ ^18^, ^19^, ^20^, ^21^, ^22^, ^23^ ^]^ It is thus interesting to ask whether CoVO_3_‐I is a Kitaev magnet too. To address this question, we performed DFT+U+SO calculations in two different modes that enabled or suppressed the Co–V interactions, respectively, see SI for further details. The exchange couplings obtained for the first set reveal a strong deformation of the magnetic honeycomb lattice that stabilizes, without frustration, the Co zigzag‐type collinear magnetic order as experimentally observed, see **Figure** **4**a. The second set stabilizes a ferromagnetic in‐plane order, which is observed in the sibling compound CoTiO_3_ where the second 3*d* ion is nonmagnetic Ti^4+^.^[^ ^24^, ^25^ ^]^ Thus, the presence of V^4+^ has a strong influence on the exchange couplings in the Co honeycomb layers. Even though V^4+^ ions develop a partial pairing and in principle should be nonmagnetic, they exhibit a perpendicular ordered moment in our diffraction data to avoid frustration with the Co sublattice.

In CoVO_3_‐II, both Co and V atoms independently form *hcp* lattices, which are nonfrustrated unless interactions beyond nearest neighbors are taken into consideration. Our calculations suggest that such long‐range interactions are negligible, but dissimilar nearest‐neighbor interactions are observed. While the Co sublattice is antiferromagnetic with *J*_Co_ = 17 K, the V sublattice is ferromagnetic with *J*_V_ = −25 K. Any coupling between such incompatible sublattices would necessarily lead to frustration and prevent magnetic ordering in both. Therefore, we interpret the formation of a spin glass in CoVO_3_‐II as the effect of two dissimilar magnetic sublattices present in this compound. It is an interesting example of a well cation ordered compound with absence of magnetic order due to intrinsic competition between the dissimilar magnetic sublattices but unrelated to competing electronic instabilities or dimerization effects as in other oxides.

The discovery of the higher‐pressure polymorph of CoVO_3_ is also remarkable as among A‐site containing Co oxides only a handful of examples are known to crystallize in the ABO_3_ corundum‐ or perovskite‐type derivatives, namely: CoTeO_3_, Co_2_CoTeO_6_, Co_2_InSbO_6_ and Co_2_ScSbO_6_^[^ ^26^, ^27^, ^28^, ^29^, ^30^ ^]^ and it points out to a novel field of “A‐site cobaltites” obtained via HP‐HT conditions.

## 3 Conclusion

The discovery of CoVO_3_‐II establishes the first compound adopting the LiNbO_3_‐type structure stabilized with two 3*d* magnetic transition metals. It presents a spin‐glass behavior that results from Co‐V interactions and thus materializes an interesting example of a compound that is magnetically disordered due to intrinsic competition between the dissimilar magnetic sublattices. On the contrary, the lower pressure polymorph CoVO_3_‐I presents a distorted ilmenite‐type structure with Co spins ordered in a zigzag‐type antiferromagnetic arrangement within the honeycomb layers, as supported by DFT. First‐principle calculations indicate that the V influence on this magnetic structure is crucial, as a FM structure known for CoTiO_3_ would be stabilized otherwise.

After preparation of this manuscript, but prior to its submission, it came to our attention that a neutron diffraction study of CoVO_3_‐I has been carried out by H. Yamamoto et al.^[^ ^31^ ^]^ They report the same magnetic order in the Co sublattice as in our work, but its origin was not revealed.

## Conflict of Interest

The authors declare no conflict of interest.

## Supporting information

Supporting Information

## Data Availability

The data that support the findings of this study are available from the corresponding author upon reasonable request.
