# Supplementary material for: CoVO3 High‐Pressure Polymorphs: To Order or Not to Order?
Source: Adv Sci (Weinh). 2023 Dec 16;11(9):2307766. doi: 10.1002/advs.202307766 (PMC10916632; doi:10.1002/advs.202307766)
Supplement: Supplementary file 1 — Supporting Information [file ADVS-11-2307766-s001.pdf]

ADVANCED SCIENCE
Open Access

Supporting Information

for *Adv. Sci.*, DOI 10.1002/advs.202307766

$CoVO_3$ High-Pressure Polymorphs: To Order or Not to Order?

Elena Solana-Madruga, Olivier Mentré, Alexander A. Tsirlin, Marielle Huvé, Dmitry Khalyavin, Clemens Ritter and Angel Moisés Arévalo-López**

$CoVO_3$ high-pressure polymorphs: To order or not to order?

Elena Solana-Madruga, [a] [b] Olivier Mentré, [a] Alexander A. Tsirlin, [c] Marielle Huvé, [a] Dmitry Khalyavin, [d] Clemens Ritter [e] and Angel Moisés Arévalo-López. *[a]

Supporting Information

Structural characterisation

$CoVO_3$-I and II crystal structures have been fully characterised using the combination of single crystal, synchrotron (SXRD) data collected in MSPD at ALBA and neutron (NPD) powder diffraction data collected in D20 at the Institut Laue-Langevin and WISH at ISIS. Final refinements for $CoVO_3$-I included in the main text correspond to WISH data. Further data, fits and structural features are included in Figures S1-S5 and Tables S2-S6.

High temperature SXRD has been performed on $CoVO_3$-I, which starts transforming into the non-distorted ilmenite structure with *R-3* space group above 500 K (Figure S1).

Single crystal and electron diffraction were collected in $CoVO_3$-II at room temperature. They agree with the presence of a *c*-glide plane suggesting either *R-3c* or *R3c.* Single crystal data refined better in the non-centrosymmetric space group and the M – O distances agreed well for Co^{2+} and V^{4+}, see Figure S2 and Table S1.

In all cases, structural model refinements were performed using the FullProf suite package.[1] Pseudo-Voigt and a convolution pseudo-Voigt with back-to-back exponential functions were used to fit powder diffraction peak shapes for SXRD and D20 data and for WISH data, respectively, while background was refined using an interpolation function between experimental points. Magnetic symmetry analysis for $CoVO_3$-I used BasIreps tool implemented in FullProf and ISODISTORT to obtain the magnetic symmetry [2,3].

Supporting figures

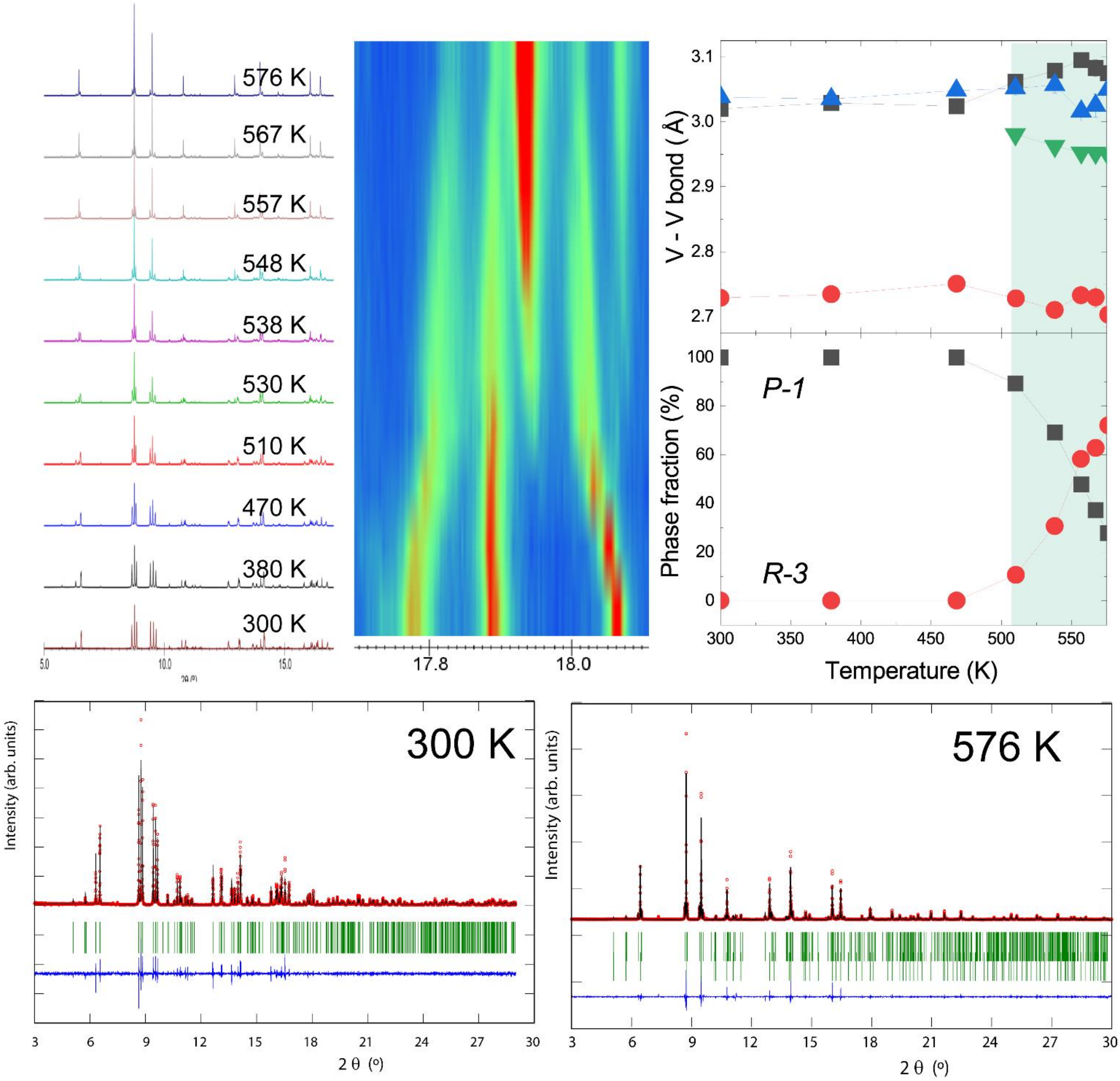

Figure S1. SXRD data collected upon heating from 300 K to 576 K for $CoVO_3$-I along with the V – V bond distances and the phase fraction. The lower panel shows Rietveld fits of the triclinic distortion observed at room temperature with *P-1* space group (left) and the rhombohedral non-distorted *R-3* ilmenite structure observed at high temperatures (right).

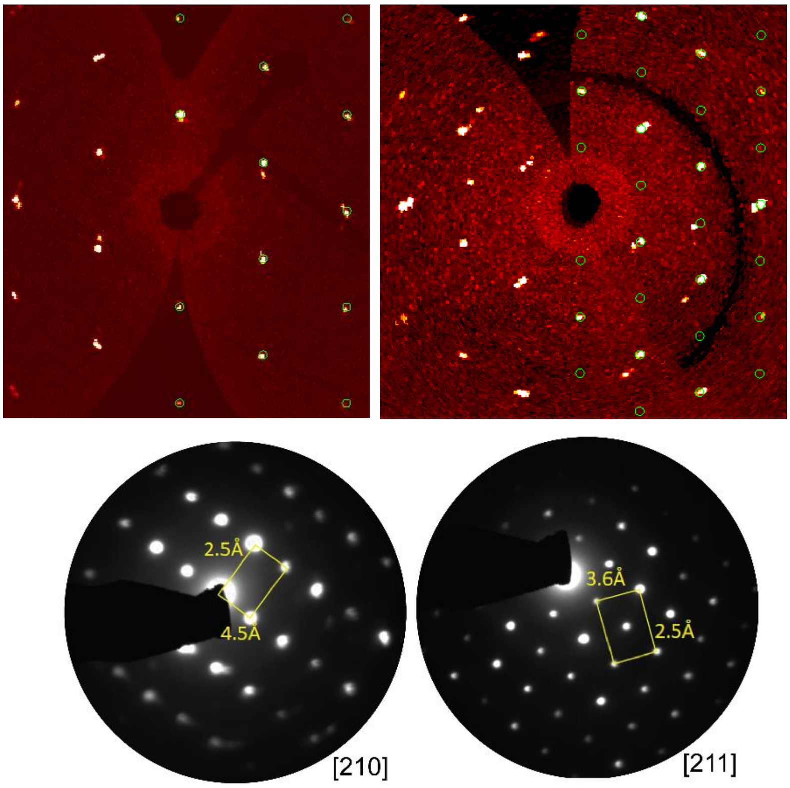

Figure. S2. Top. (hk0) and (h0l) planes of $CoVO_3$-II from single crystal XRD data. Reflection conditions in (*h*-*h*0*l*): *h*+*l* = *3n* and *l* = *2n* agree with the presence of a *c*-glide plane, thus suggesting either *R3c* or *R-3c* symmetry. Bottom. Selected area electron microscopy of $CoVO_3$-II along the [210] and [211], the absence of diffuse scattering points out to a well-ordered structure.

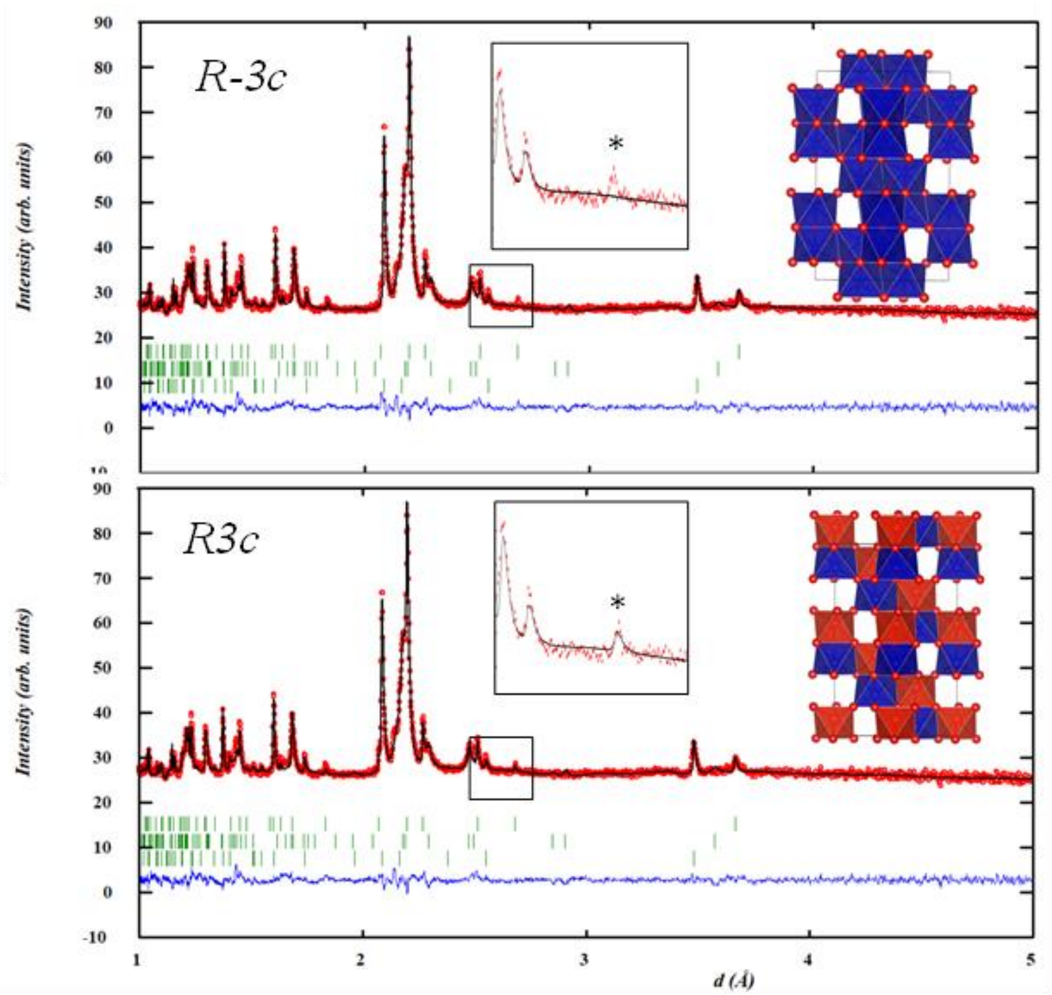

Figure. S3. Comparison of the corundum *R-3c* (top) and $LiNbO_3$-type *R3c* (bottom) structural models inferred from single crystal XRD for $CoVO_3$-II from their Rietveld fits against NPD data collected at WISH. The absence of the inversion symmetry provides intensity to the (104) peak at 2.68 Å as shown in the enlarged regions (inset). The impossibility to reproduce this intensity in the top model confirms the LN -type structure for $CoVO_3$-II.

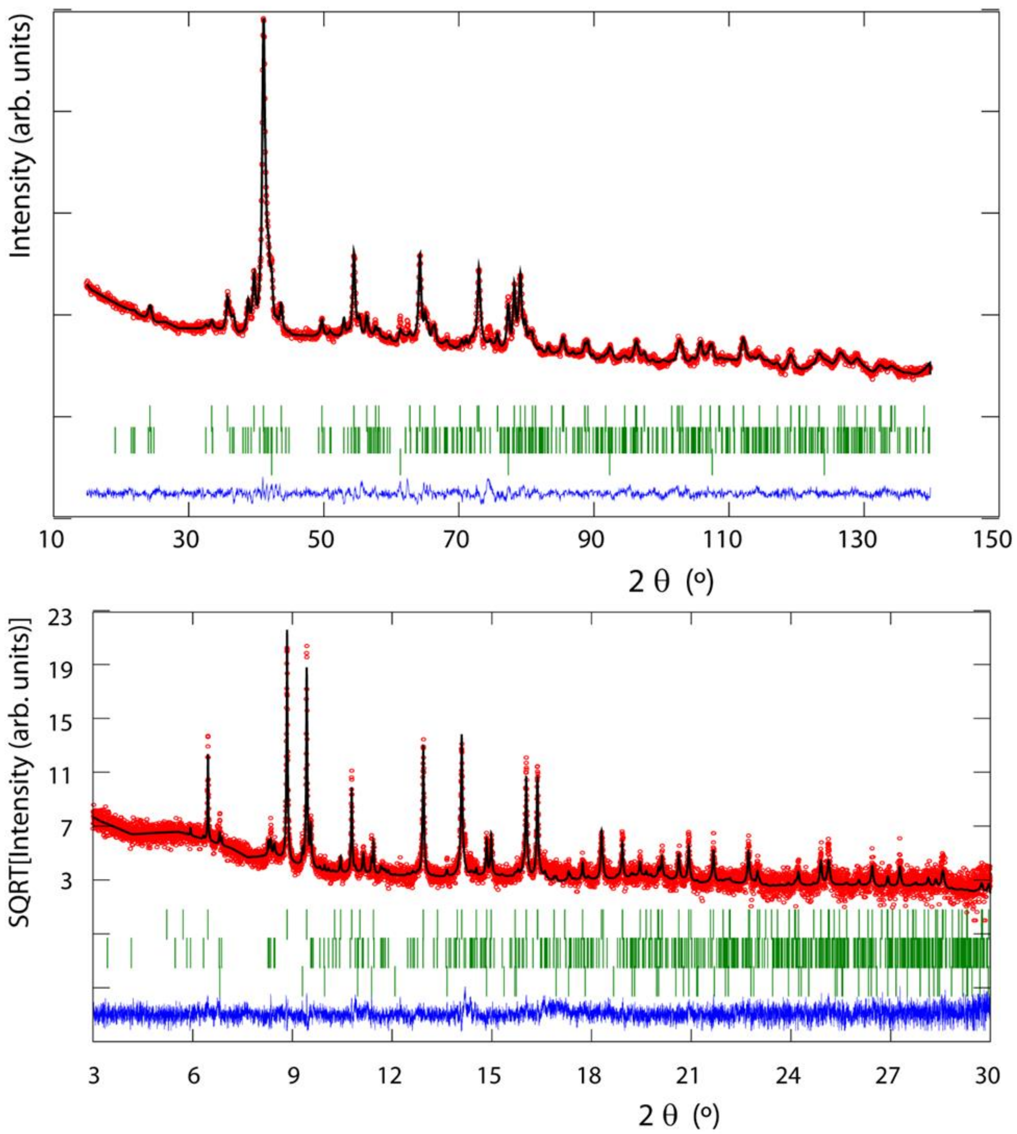

Figure. S4. Rietveld fits of the $LiNbO_3$-type *R3c* structural model of $CoVO_3$-II from the combined refinement against NPD data collected at 130 K in D20 (λ = 1.54 Å, 90º take off angle) (top) and SXRD data collected at 300 K in BL04-MSPD at ALBA (λ = 0.4138 Å) (bottom).

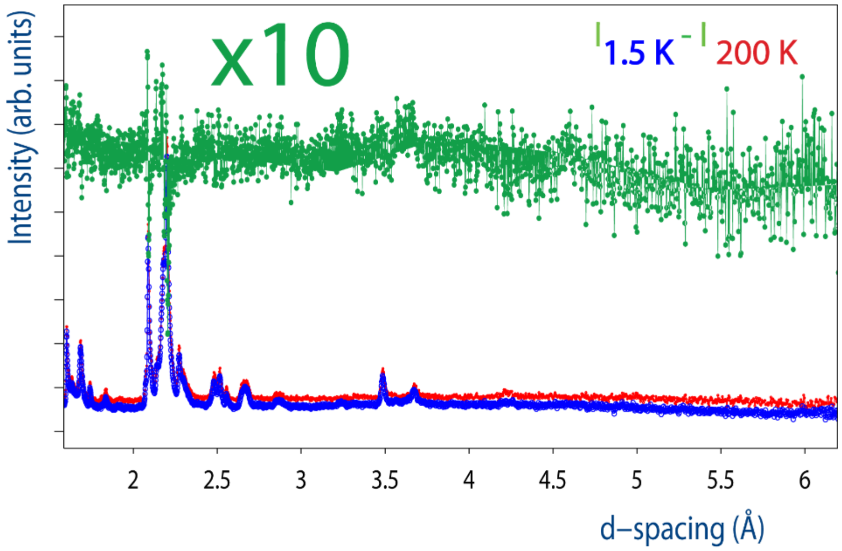

Figure. S5. Difference pattern between 1.5 and 200 K data collected for $CoVO_3$-II at WISH on a 16 mg sample. Only short-range correlations are distinguishable.

DFT Calculations

Density-functional-theory (DFT) band-structure calculations were performed in the VASP [4,5] and FPLO [6] codes with the Perdew-Burke-Ernzerhof (PBE) version of the exchange-correlation potential [7]. Experimental lattice parameters and atomic positions were used without further optimization. Magnetic exchange couplings were extracted by a mapping procedure [8] from total energies of collinear and non-collinear spin configurations obtained on the DFT+*U*+SO level to account for electronic correlations in the Co and V 3*d* shells. The DFT+*U*+SO parameters were set to U_d = 5 eV (on-site Coulomb repulsion) and J_d = 1 eV (Hund's coupling) with the double-counting correction in the atomic limit [9,10]. The exchange couplings enter the spin Hamiltonian

$$H = \sum_{<ij>} J_{ij}^{\mathrm{Co}} \boldsymbol{S_i S_j} + \sum_{<ij>} J_{ij}^{\mathrm{V}} \boldsymbol{s_i s_j} + \sum_{<ij>} J_{ij}^{\mathrm{Co-V}} \boldsymbol{S_i s_j}$$

where S_i = 3/2 (Co^{2+}) and s_i = ½ (V^{4+}). Note that only the first term remains in $CoVO_3$-I where the V^{4+} ions are nonmagnetic.

Comparison between the two polymorphs

Figure S6 compares PBE density of states for both polymorphs of $CoVO_3$. They show very similar oxygen bands. Moreover, the bands at the Fermi level combine Co 3*d* and V 3*d* states, suggesting both Co and V as possible magnetic ions. The main difference between the two polymorphs is seen in the V 3*d* bands above the Fermi level. In $CoVO_3$-I, the V e_g bands at 2.5-4.0 eV split into four narrow peaks that are reminiscent of molecular orbitals. On the other hand, $CoVO_3$-II shows a single and uniform e_g band complex. This difference parallels the formation of V-V dimers in $CoVO_3$-I but not in $CoVO_3$-II.

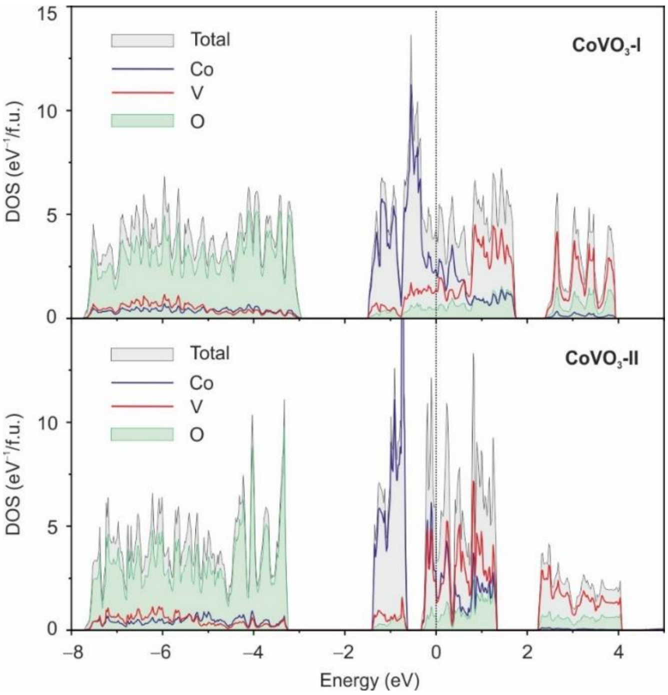

Figure. S6. PBE density of states for both polymorphs of $CoVO_3$. The Fermi level is at zero energy. The spectra are metallic because electronic correlations were not included. DFT+*U*+SO calculations produce insulating states with the band gap of about 1 eV.

DFT+*U*+SO calculations yield the magnetic moment of about 3 μ_B on Co and confirm the valence state of Co^{2+} in agreement with the experiment. Smaller magnetic moments of about 1 μ_B are found on vanadium as expected for V^{4+}. Since spin configurations in DFT+*U*+SO are classical in nature, the singlet, fully nonmagnetic state of the V-V dimers in $CoVO_3$-I cannot be reproduced in the calculation. However, spin configurations with antiparallel spins on vanadium were found to be more energetically favorable than ferromagnetic ones, thus confirming the dimer formation.

- **$CoVO_3$-I**

In $CoVO_3$-I, the Co atoms form a honeycomb network with three dissimilar nearest-neighbor exchange pathways (J_1) that we label with X, Y, and Z. A similar separation into the nonequivalent X-, Y-, and Z-pathways is found for the next-nearest-neighbor couplings J_2 (Figure S7). Table S1 lists calculated exchange couplings for $CoVO_3$-I and also includes the interlayer coupling $J_\perp$ that corresponds to the shortest Co-Co distance between the honeycomb layers. Exchange couplings were calculated for two different scenarios. In the first one, same quantization axis was chosen for V and Co, such that their magnetic moments were either parallel or antiparallel to each other. In the second set of calculations, V spins were set orthogonal to the Co spins, such that Co-V interactions are suppressed.

Table S1. Calculated exchange couplings for $CoVO_3$-I. The first set of the J_i values was obtained with the same (parallel or antiparallel) direction of the Co and V spins. The second set of the J_i values was obtained with the orthogonal Co and V spins to exclude Co-V interactions.

	Co-Co distance (Å)	J_i (K), set 1	J_i (K), set 2
J_{1X}	2.753	50	–10
J_{1Y}	2.846	–75	–10
J_{1Z}	3.150	–64	–8
J_{2X}	4.918	31	2
J_{2Y}	4.985	29	–1
J_{2Z}	5.048	–32	0
$J_{\perp}$	4.180	–10	–2

The exchange couplings from the first set of the calculations favor zigzag-type collinear magnetic order, which is quite similar to the experimental magnetic structure of $CoVO_3$-I. The zigzag order is stabilized by a strong deformation of the honeycomb lattice with antiferromagnetic J_{1X} but ferromagnetic J_{1Y} and J_{1Z}. Likewise, we find two antiferromagnetic (J_{2X}, J_{2Y}) and one ferromagnetic (J_{2Z}) second-neighbor coupling. This interplay of ferromagnetic and antiferromagnetic couplings is also seen in the experimental value of the Curie-Weiss temperature (θ = –45 K), which is about 3 times smaller than T_N = 141 K. Indeed, θ is a simple sum of the exchange couplings and becomes small when ferromagnetic couplings are added to antiferromagnetic ones, whereas T_N depends on the magnitude of the exchange couplings that stabilize zigzag order, regardless of their sign.

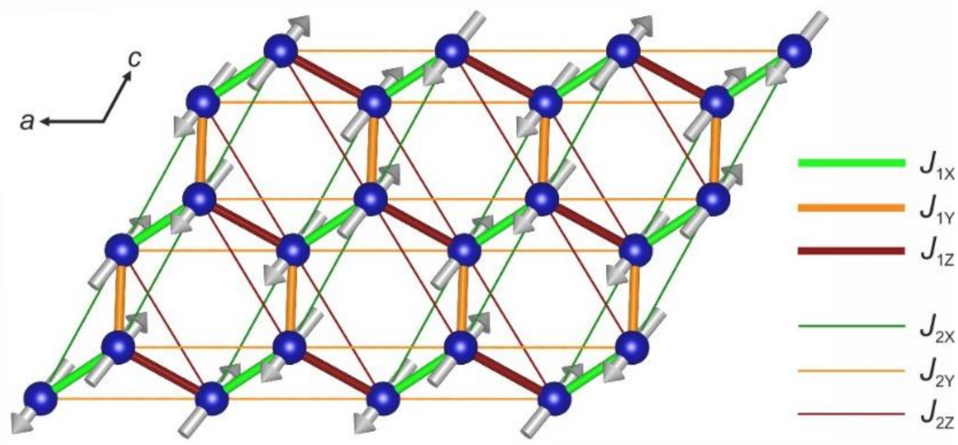

Figure S7. Honeycomb network of Co atoms in $CoVO_3$-I. The gray arrows show the zigzag-type collinear order favored by the first set of exchange couplings from Table A1 (ferromagnetic J_{1Y}, J_{1Z}, J_{2Z} and antiferromagnetic J_{1X}, J_{2X}, J_{2Y}).

Interestingly, the second set of calculations produces a very different magnetic model. Ferromagnetic nearest-neighbor interactions and weak antiferromagnetic second-neighbor interactions found in this case would stabilize ferromagnetic order, which is indeed observed in the sibling compound $CoTiO_3$ [11]. We conclude that the presence of V^{4+} with its partially filled *d*-states near the Fermi level has a strong influence on the exchange couplings in the Co honeycomb layers, even though V^{4+} ions are bound into dimers and nonmagnetic.

- **$CoVO_3$-II**

In $CoVO_3$-II, both Co and V atoms form an hcp lattice, which is nonfrustrated for nearest-neighbor interactions and becomes frustrated when interactions beyond nearest neighbors are included. Our calculations suggest that each of the Co and V sublattices is almost nonfrustrated, with the nearest-neighbor couplings of J_{1Co} = 17 K and J_{1V} = –25 K (metal-metal distance of 3.684 Å), as well as the second- and third-neighbor couplings of about 2 K (metal-metal distances of 5.027 Å and 5.387 Å, respectively). Interestingly, the Co sublattice is antiferromagnetic, whereas the V sublattice is ferromagnetic. Any coupling between them necessarily leads to a frustration and should prevent magnetic order in both. We estimate the average Co-V coupling, $J_{Co\text{-}V}$ = 4 K as the subleading energy scale in the system. Therefore, we interpret the formation of spin glass in $CoVO_3$-II as the effect of two dissimilar magnetic sublattices present in this compound (Figure S8).

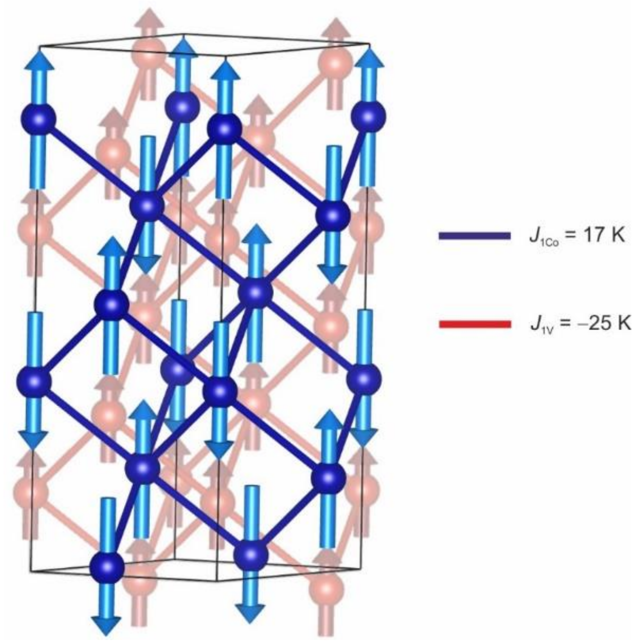

Fig. S8. The structure of $CoVO_3$-II with the interpenetrating magnetic sublattices of Co^{2+} (antiferromagnetic) and V^{4+} (ferromagnetic).

Supporting tables

Table S2. Crystal data and structure refinement information for $CoVO_3$-II LN-type phase from single crystal diffraction collected at 300 K. Space group *R3c*.

Molar mass (g mol^{-1})	157.9

Unit cell dimensions (Å)	a = 5.008(5), c = 13.54(1)
Unit cell angles (°)	α = 90º, β = 90º and γ = 120º
Unit cell volume (Å^3)	293.96(3)
Wavelength	Mo $K\alpha$ (0.71073 Å)
Absorption coefficient (mm^{-1})	12.874
F(000)	444
Θ range / °	5.585 to 30.831
hkl range	$-7 \le h \le 3$, $0 \le k \le 7$, $0 \le l \le 18$
Total no. reflections	181
Independent reflections	99
Data/parameters	99 / 17
Goodness of fit on F^2	2.35
R1/wR2 (I ≥ 3σ)	0.0297/0.0374
R1/wR2 (All data)	0.0334/0.0379
Largest diff. peak and hole (e^- A^3)	0.61 / -1.13

Table S3. Structural details from single crystal data at 300 K for $CoVO_3$-II. R_p = 0.91/15.3%, R_{wp} = 1.18/19.7 %, R_B = 3.89%, R_f = 2.59%, χ^2 = 2.49/1.32.

Site	x	y	z	Occ	B_{iso} (Å^2)
Co (6a)*	0.0	0.0	0.0612	1	0.5(1)
V (6a)	0.666	0.333	0.1043(4)	1	0.8(2)
O (18b)	0.7067(9)	0.677(1)	0.167(1)	1	1.5(1)

* Used as a cell reference.

Table S4. M – O and M – M distances from single crystal data.

Site	x	y	z	Occ	B_{iso} (Å^2)
Co (6a)*	0.0	0.0	0.0612	1	0.5(1)
V (6a)	0.666	0.333	0.1043(4)	1	0.8(2)
O (18b)	0.7067(9)	0.677(1)	0.167(1)	1	1.5(1)

* Used as a cell reference.

Table S5. Main structural details from a combined refinement against 300 K SXRD data and 130 K NPD collected at D20 for the $CoVO_3$-II polymorph. Space group *R3c* and cell parameters *a* = 5.0172(4) Å and *c* = 13.610(1) Å. Agreement factors (NPD/SXRD) R_p = 0.91/15.3%, R_{wp} = 1.18/19.7 %, R_B = 3.89%, R_f = 2.59%, χ^2 = 2.49/1.32.

Site	x	y	z	Occ	B_{iso} (Å^2)
Co (6a)*	0.0	0.0	0.0612	1	0.5(1)
V (6a)	0.666	0.333	0.1043(4)	1	0.8(2)
O (18b)	0.7067(9)	0.677(1)	0.167(1)	1	1.5(1)

* Used as a cell reference.

Table S6. Main structural details from the Rietveld fit of $CoVO_3$-I distorted ilmenite structure against 160 K NPD collected at WISH Space group *P-1* and cell parameters *a* = 4.9999(4) Å, *b* = 5.4353(4) Å, *c* = 4.9463(5) Å, α = 90.10(1)º, β = 119.79(1)º and γ = 63.40(1)º. Agreement factors R_p = 4.36%, R_{wp} = 4.79 %, R_B = 7.14%, R_f = 8.20%, χ^2 = 1.53 (selected from the middle bank as representative).

Site	X	Y	z	Occ	B_{iso} (Å^2)*^{2}
Co (2i)	0.288(5)	0.554(3)	0.660(5)	1	1.1(3)
V (2i)*	0.7272	0.945	0.8591	1	0.51
O1 (2i)	0.182(2)	0.741(2)	0.237(1)	1	1.0(1)
O2 (2i)	0.492(2)	0.772(2)	0.954(2)	1	1.0(1)
O3 (2i)	0.801(2)	0.777(2)	0.583(2)	1	1.0(1)

* Used as a cell reference. *^{2} B_{iso} values constrained for all oxygen atoms for data collected on a small 54 mg sample.

[1] J. Rodriguez-Carvajal, *Physica B* (1993) 192, 55.
[2] H. T. Stokes, D. M. Hatch, B. J. Campbell, ISODISTORT, ISOTROPY Software Suite, iso.byu.edu.
[3] B. J. Campbell, H. T. Stokes, D. E. Tanner, D. M. Hatch, *J. Appl. Cryst.* (2006) 39, 607-614.
[4] G. Kresse, J. Furthmüller, *Computational Materials Science* (1996), 6, 15-50.
[5] G. Kresse, J. Furthmüller, *Phys. Rev. B* (1996), 54, 11169.
[6] K. Koepernik, H. Eschrig, *Phys. Rev. B* (1999), 59, 1743.
[7] J. P. Perdew, K. Burke, M. Ernzerhof, *Phys. Rev. Lett*. (1996),77, 3865.
[8] H. J. Xiang, E. J. Kan, Su-Huai Wei, M.-H. Whangbo, X. G. Gong, *Phys. Rev. B* (2011), 84, 224429.
[9] A. A. Tsirlin, R. Nath, J. Sichelschmidt, Y. Skourski, C. Geibel, H. Rosner, *Phys. Rev. B* (2011), 83, 144412.
[10] D. L. Quintero-Castro, G. J. Nilsen, K. Meier-Kirchner, A. Benitez-Castro, G. Guenther, T. Sakakibara, M. Tokunaga, C. Agu, I. Mandal, A. A. Tsirlin. *Phys. Rev. M* (2023), 7, 045003.
[11] R. E. Newnham, J. H. Fang, R. P. Santoro *Acta. Cryst.* (1964), 17, 240.
